# Community perspectives on COVID-19 vaccine allocation ethical principles in Uganda: a cross-sectional study

**DOI:** 10.3389/fpubh.2026.1755524

**Published:** 2026-02-02

**Authors:** Juliet Kiguli, Lesley Rose Ninsiima, Stuart Ssebibubbu, Tom Okade, Ramadhan Kirunda, Celia Nalwadda, Joyce Nabaliisa, John Mary Mooka Kamweri

**Affiliations:** 1 Department of Community Health and Behavioral Sciences, Makerere University, Kampala, Uganda; 2 Department of Diseases Control and Environmental Health, Makerere University, Kampala, Uganda; 3 Uganda National Academy of Sciences, Makerere University, Kampala, Uganda; 4 Institute of Ethics, Uganda Martyrs University, Kampala, Uganda

**Keywords:** COVID-19, cross sectional study, ethical principles, Uganda, vaccine allocation

## Abstract

**Background:**

The scarcity of COVID-19 vaccines raised tough questions about who should get them first. While global guidelines stress fairness, they may overlook local realities and community voices. Without understanding these perspectives, efforts to ensure equity and build trust risk falling short. Therefore, this study aimed to assess the multifaceted perceptions, knowledge, and attitudes of community influencers in Uganda regarding COVID-19 vaccination.

**Methods:**

This study adopted a mixed-methods cross-sectional descriptive design. The study participants were identified through community-based organizations (CBOs) to get a broad representation of the various community members and practical community entry points. Quantitative data were analyzed using the Statistical Package for Social Sciences (SPSS). Qualitative data were analyzed using thematic content analysis with NVivo 12.0 software.

**Results:**

A total of 100 Ugandan participants took part in the study, less than half of the participants 46.0% knew Uganda/MoH guidelines only. Forty two percent (42%) were neutral on vaccines curbing COVID-19 spread, with uniform effectiveness beliefs (*p* > 0.05). Ninety percent (90%) supported guiding principles. Only few of the participants 31% agreed that they were very likely to contract COVID-19 after vaccination, 81% favored equal treatment but low trust in equity across classes or tribes. From the qualitative data, five themes which included, gender/power imbalances, economic divides, social norms, herbal alternatives, and tribalism. Global equity skepticism was high (57% no trust in LMIC/HIC quality parity); 86% endorsed national equity, but doubts persisted on at-risk prioritization and fair distribution.

**Conclusion:**

While awareness of guidelines is relatively high, it is overshadowed by pervasive skepticism regarding vaccine efficacy, profound distrust in the fairness and integrity of distribution processes, and a feeling of exclusion from decision-making at global and national levels. Therefore, addressing these challenges requires a multi-pronged approach that goes beyond simply disseminating information about COVID-19 vaccination. With the strong support for ethical principles in COVID-19 vaccination, addressing equity gaps through culturally attuned strategies is essential for equitable distribution and sustained public trust in Uganda.

## Introduction

The coronavirus disease (COVID-19) pandemic posed unprecedented challenges to global health systems, necessitating the rapid development and distribution of vaccines to control the spread of the virus (1, 2). In December 2019, China reported 44 “Pneumonia of unknown cause” cases to the World Health Organization (WHO) (3, 4). Later, in January 2020, this disease that later came to be known as coronavirus disease 2019 (COVID-19) was declared “a Public Health Emergency of International Concern” (5). By July 2020, 369,928 infections in Africa had occurred, and 6,974 had died (6).

In Uganda, a country in East Africa with a population of approximately 47 million, the pandemic exacerbated existing health inequities, straining an already overburdened healthcare system and highlighting vulnerabilities in rural and urban communities (7). According to the Uganda presidential address in June and July 2020, around the same time, the country had registered 953 cases of infection, 892 recoveries, and no deaths (8). As of 31 January 2021, Kenya had 100,773 cases and 1,763 deaths (9). In response to the COVID-19 pandemic, the Governments of Kenya and Uganda adopted similar public health measures to contain its spread. The rapid development of COVID-19 vaccines marked a turning point in the global response, offering a critical tool for mitigating transmission, reducing severe illness, and saving lives. Some initial measures included refusing to repatriate citizens studying in China, mandatory institutional quarantine, physical and social distancing (10).

In Uganda, similar to other countries, the ethical principles governing vaccine allocation have become a focal point of discussion. Equity, justice, and community engagement principles are particularly salient in this context, as they directly influence public acceptance and the overall effectiveness of vaccination campaigns. The ethical allocation of vaccines is not merely a logistical challenge but also a moral imperative, especially in resource-limited settings where disparities in healthcare access are pronounced (11, 12). However, the initial scarcity of vaccines necessitated difficult decisions on allocation, raising complex ethical questions about fairness, equity, and prioritization.

Ethical frameworks for vaccine allocation were proposed by international bodies and national advisory committees to guide these decisions. The concept of beneficence, which emphasizes the importance of acting in the best interest of individuals and communities, is central to the ethical discourse surrounding vaccine distribution. Vaccination programs must not only aim to achieve herd immunity but also ensure that vulnerable populations are prioritized (13). Additional values emphasized in global discussions encompass helping those with the greatest need, reducing health disparities, rewarding instrumental value (such as protecting frontline healthcare workers who enable societal functioning), and prioritizing the worst-off to foster reciprocity and social utility. In Uganda, where healthcare resources are limited, the challenge lies in balancing the need for rapid vaccine deployment with the ethical obligation to ensure equitable access for all segments of the population, particularly marginalized groups who may face barriers to healthcare (14).

The WHO guidelines on vaccine allocation underscore the need for a values-based framework that prioritizes health workers and high-risk populations, reflecting a commitment to equity and justice in public health (11). The vaccine rollout began in March 2021 with limited doses primarily sourced through COVAX and bilateral donations, leading to challenges in equitable distribution. However, there is a gap in understanding broader community views on the ethical underpinnings of allocation, particularly in diverse settings where sociocultural factors may influence acceptance.

The WHO Values Sage Framework offers guidance globally on the allocation of COVID-19 vaccines between countries and offers guidance nationally on the prioritization of groups for vaccination within countries while supply is limited (15). The framework has been developed to provide a values foundation for SAGE recommendations on priority target groups for specific COVID-19 vaccines at different stages of supply availability. It intends to be a helpful tool for policymakers and expert advisors at the global, regional, and national levels as they make allocation and prioritization decisions about COVID-19 vaccines. In addition, the framework is intended to help all stakeholders, including community and advocacy groups, the general public, health professionals, and other civil society organizations, as they contribute to decisions about how limited supplies of COVID-19 vaccines should be deployed for optimal impact. The framework addresses only ethical issues regarding allocating and prioritizing COVID-19 vaccines. As such, the framework articulates the overall goal of COVID-19 vaccine deployment and provides six core principles that should guide the distribution of vaccines (15).

Community perspectives play a crucial role in shaping the ethical landscape of vaccine allocation. Engaging with local communities to understand their concerns, beliefs, and values regarding vaccination can enhance trust and acceptance of the vaccine. Top-down approaches that overlook local perspectives risk perpetuating inequities and undermining public health efforts. In Uganda, historical factors, including past injustices in healthcare, may contribute to vaccine hesitancy, making it imperative for health authorities to adopt a participatory approach incorporating community voices in decision-making processes (16). Additionally, this approach fosters a sense of ownership among community members, helps address misinformation, and builds confidence in vaccination efforts. This study addresses this gap by exploring the community members' perspectives, including their feelings, concerns, attitudes, agreement, and underlying reasoning, regarding the ethical principles and prioritization criteria for COVID-19 vaccine distribution.

## Methods

### Study design and setting

This study adopted a mixed-methods, cross-sectional descriptive design to explore community perspectives on COVID-19 vaccine allocation ethical principles in Uganda between months of June and August 2021. The design incorporated both quantitative and qualitative approaches to provide a comprehensive understanding of perceptions, motives, and feelings among community members.

The study areas were divided into four cultural regions as follows:

- Central Uganda region including the Buganda Central and Buganda South;
- Eastern Uganda Region including Teso, Busoga, Bukedi, Bugisu, Sebei;
- Western Uganda including Kigezi, Ankole, Toro, and
- Northern Uganda including Langi, Karamojong, Lugbara, and Acholi.

### Study population and selection criteria

The study population was identified through community-based organizations (CBOs) to get a broad representation of the various community members and practical community entry points. These CBOs focused on cultural, social, health initiatives and economic empowerment. The recruitment and sampling process was led by the CACVAEP study coordinator, who obtained a list of community-based organizations from the civil society organization network (CSO) focal person within the region with approximately 35% Central, 30% Eastern, 25% Western, and 10% Northern. Later, individuals from the CBOs were categorized into four major groups including women, men, youth and special groups such as people living with disability, people living with HIV, traditional healers and village elders. Sample size was therefore determined pragmatically by the need to ensure adequate representation from each of the four cultural regions (Central, Eastern, Western, and Northern Uganda) and to capture diverse perspectives. This flexible approach allowed adjustment for logistical accessibility and ensured sufficient participants per region. Within each group, study participants were purposively sampled across the various groups and met the following inclusion criteria: aged 18 years and above; owned a smartphone or laptop; knew the national languages; consented to participate in the survey.

### Data collection process

Data captured included engagement of community members in understanding their perceptions of COVID-19 vaccine allocation ethical principles, which are relevant for ensuring equitable access and fair allocation of vaccines, therapeutics, and diagnostics. The principles included:

Human wellbeing: protect and promote human wellbeing, including health, social and economic security, human rights and civil liberties, and child development. The principle further requires that those making vaccine allocation and prioritization decisions determine what vaccine deployment strategies will best promote and protect all the implicated dimensions of wellbeing, including strategies for containing transmission, reducing severe disease (including long-term sequelae) and death, or a combination.

Equal respect: recognize and treat all human beings with equal moral status and their interests as deserving of equal moral consideration. The principle that all people should be treated as moral equals, entitled to equal respect and consideration of their interests, is enshrined in the Universal Declaration of Human Rights31 and the constitutional documents of many countries. Equal respect is also generally understood to be a foundational principle of ethics and of justice or equity in particular.

Global equity: ensure equity in vaccine access and benefit globally among people in all countries, particularly those in low-and middle-income countries. Countries and territories are primarily responsible for protecting and promoting the wellbeing and human rights of those living within their borders. It is thus reasonable and appropriate for countries to be concerned with securing sufficient COVID-19 vaccines to meet the needs of their populations.

National Equity: ensure equity in vaccine access and benefits within countries for groups experiencing more significant burdens from the COVID-19 pandemic. It is essential to use constrained resources efficiently, especially when the resources are of high-value such as vaccines in a devastating pandemic.

Reciprocity: honor reciprocity obligations to those individuals and groups within countries who bear significant additional risks and burdens of COVID-19 response for the benefit of society. Offering vaccines to those who take or bear exceptional risks during a pandemic, often because of their occupations, is one way to honor reciprocity obligations and express gratitude. Therefore, the principle of reciprocity should be interpreted with caution to pre-empt inappropriate claims by people and entities with disproportionate power and resources to reciprocity-based entitlement to the COVID-19 vaccine.

Legitimacy: make global decisions about vaccine allocation and national decisions about vaccine prioritization through transparent processes based on shared values, best available scientific evidence, and appropriate representation and input by affected parties. Legitimacy in the context of COVID-19 vaccines and this pandemic refers to the appropriate authority to make recommendations and governing decisions about who gets the vaccine and when. This is because different stakeholders, including different countries at the global level and interest groups at the national level, are likely to have different views about vaccine allocation and prioritization. All concerned stakeholders must know that the recommendations and decisions emanate from a legitimate body through a legitimate process.

These principles are relevant to ensuring that the allocation process enables equitable access and fair allocation of vaccines, therapeutics, and diagnostics. The data was collected using both quantitative and qualitative methods. For the quantitative approach, participants completed a closed-ended Likert scale questionnaire to investigate perceptions, motives, and feelings related to the COVID-19 vaccine ethical principles. Open-ended questions were integrated into this questionnaire for purposively selected key informants to capture richer and unstructured insights. Qualitative data were further collected through key informant interviews (KIIs) with knowledgeable individuals and an online survey administered via telephone interviews to accommodate accessibility and reach.

All processes were conducted ethically, with participant consent obtained prior to involvement. Data collection was led by the study coordinator in collaboration with regional CSO networks. No materials beyond the questionnaire and interview guides were used, and no power calculation was performed, as this was a descriptive study without hypothesis testing for effect sizes.

### Data analysis

Quantitative data were analyzed using the Statistical Package for Social Sciences (SPSS). Prior to analysis, data were reviewed and cleaned for accuracy and consistency. Descriptive statistics, including frequencies, proportions, and percentages, were used to summarize variables. To test for the significant values, bivariate analysis which was the chi-square test and one-way ANOVA test at significance level (*p* > 0.05) was done. Qualitative data were analyzed using thematic content analysis with NVivo 12.0 software. This approach complemented the quantitative findings, addressing potential biases from closed-ended questions and providing deeper insights into factors influencing vaccine acceptance. The mixed-methods integration allowed for triangulation, yielding a more comprehensive understanding of community perceptions.

## Results

### Participant characteristics

A total of 100 participants took part in the study. Most participants 24.0% were local community influencers. Most of the participants 26.0% were males aged between 30 and 49 years (Table 1).

**Table 1:** Showing the social demographics of the participants.

**No**	**Participant category**	**Frequency (*N* = 100)**	**Percentage (%)**
1	Cultural influencer	8	8.0
2	Health influencer	12	12.0
3	Local community influencer	24	24.0
4	Political influencer	4	4.0
5	Religious influencer	17	17.0
6	School influencer	13	13.0
7	Special interest (HIV, disabled, etc)	22	22.0
**Label**	**Participant age group**	**Gender**	**Percent (%)**
A	18–29	Female	13.0
		Male	12.0
B	30–49	Female	18.0
		Male	26.0
C	50+	Female	12.0
		Male	19.0

#### Knowledge, awareness and attitude towards COVID-19

Less than half of the participants 46.0% knew Uganda/MoH guidelines only (Table 2).

**Table 2:** Awareness of WHO and Uganda MOH COVID 19 vaccination guidelines.

**Awareness of WHO and National/MOH COVID guidelines**	**Percentage (%)**
Know both WHO and Uganda/MOH guidelines	36%
Know Uganda/MOH guidelines only	46%
Know WHO guidelines only	3%
Don't know either WHO or Uganda MOH guidelines	15%

#### Community Perspectives on Ethical Principles and Prioritization Criteria for COVID-19 vaccine distribution

**Figure 1:**
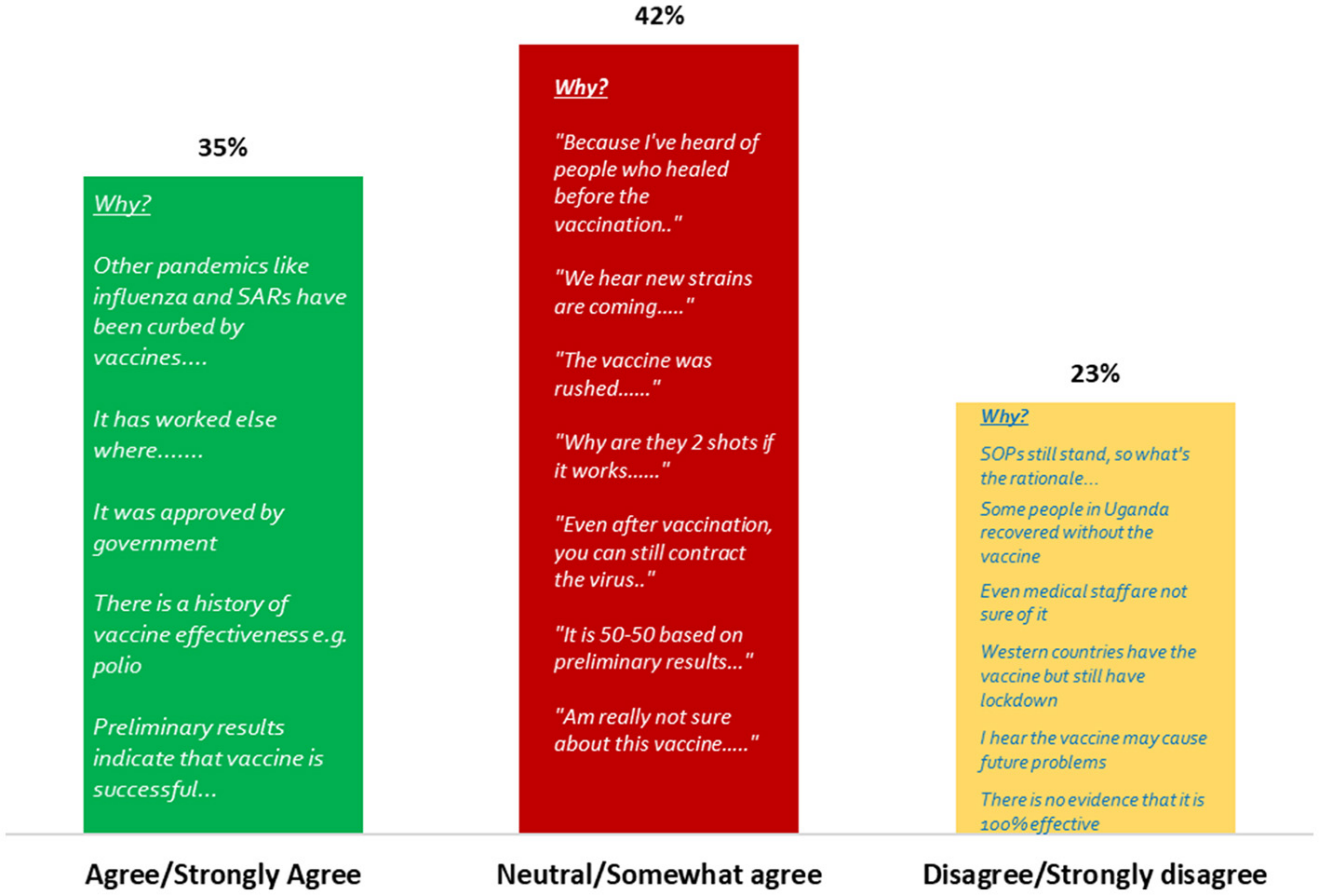
Level of agreement that vaccination can curb the spread of COVID-19 in Uganda.

Nearly half of the participants 42.0% were neutral/somewhat agreed that vaccination could curb the spread of COVID-19 in Uganda (Figure 1).

**Figure 2:**
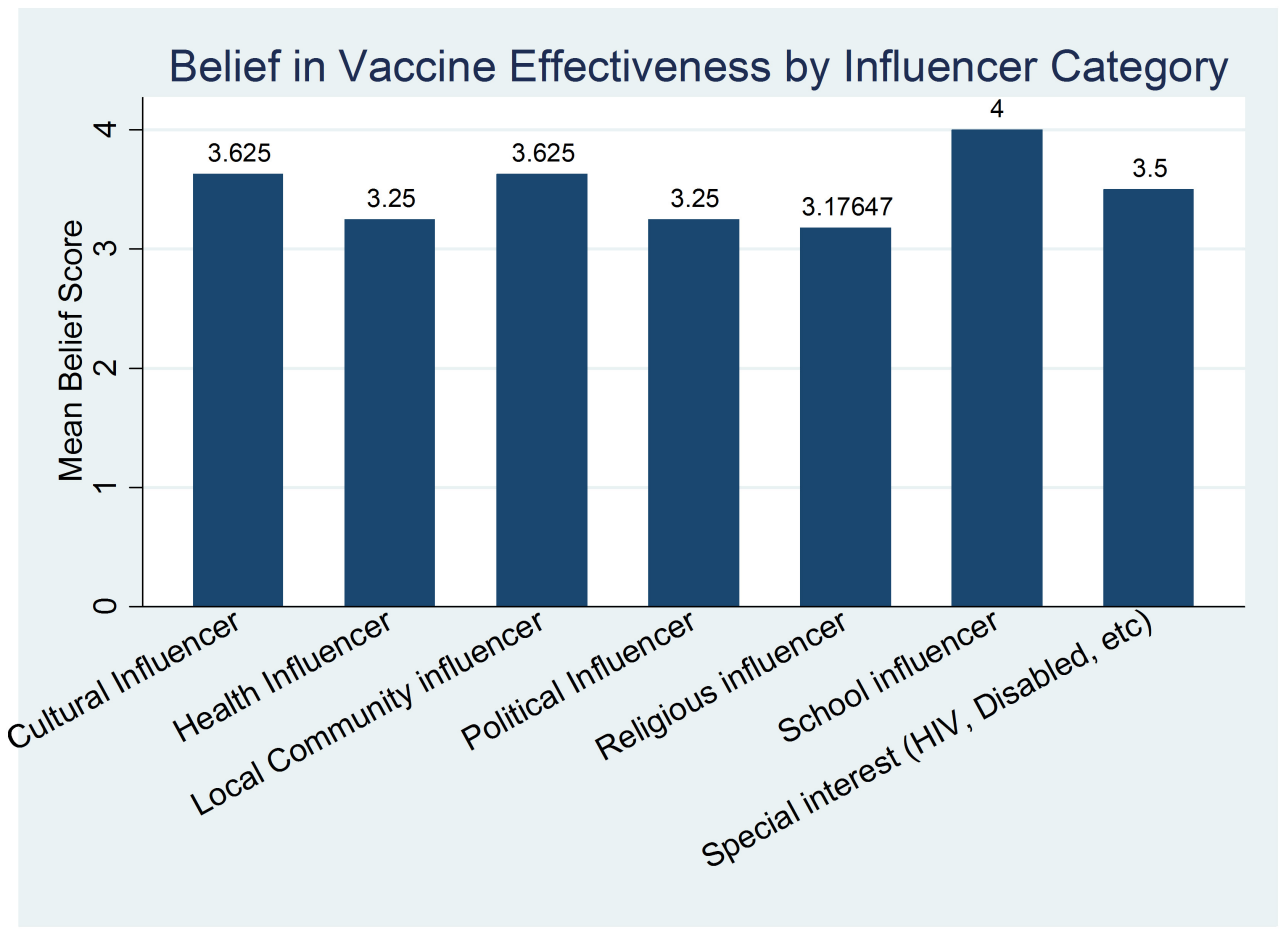
Shows the belief in vaccine effectiveness by influencer category.

An analysis on vaccine effectiveness revealed no significant variation across the participant categories. The results in Figure 2 below suggest that the participant category type have similar beliefs about vaccine effectiveness (*p* > 0.005, mean belief >3.00).

An inquiry into participants' perceptions revealed that 90% agreed there should be guiding principles for COVID-19 vaccine allocation in the country. In Table 3, a one-way ANOVA test done shows that there was no significant variation at *p* < 0.05 in the means across different participant categories.

**Table 3:** Participant category and perceptions on having vaccine guiding principles.

**Participant category**	**Summary of agreement that “we” should have guiding principles**			
	**Mean**	**Std. dev**.	**Freq**.	**% age**
Cultural influencer	3.875	0.354	8	8.0%
Health influencer	3.667	0.492	12	12.0%
Local community influencer	3.375	0.875	24	24.0%
Political influencer	3.250	1.500	4	4.0%
Religious influencer	3.471	0.800	17	17.0%
School influencer	3.846	0.376	13	13.0%
Special interest groups	3.591	0.908	22	22.0%
Total	3.570	0.782	100	100.0%

### COVID-19 vaccination ethical principles

#### Human wellbeing

**Figure 3:**
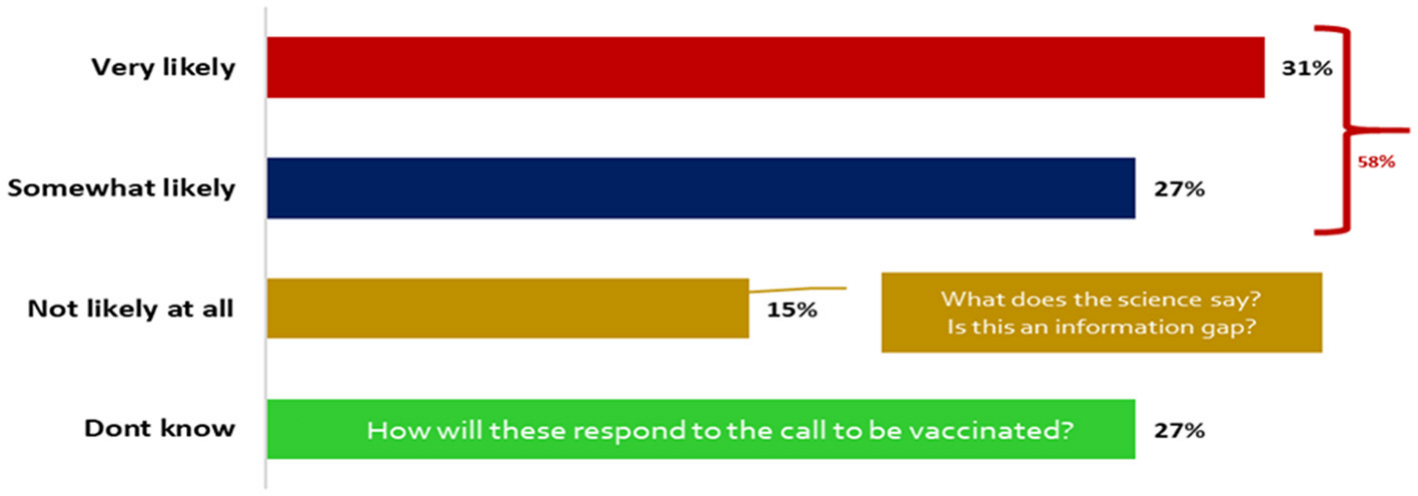
Likelihood of contracting COVID-19 after vaccination.

Only a few of the participants 31% agreed that they were very likely to contract COVID-19 after vaccination (Figure 3).

#### Equal respect

**Figure 4:**
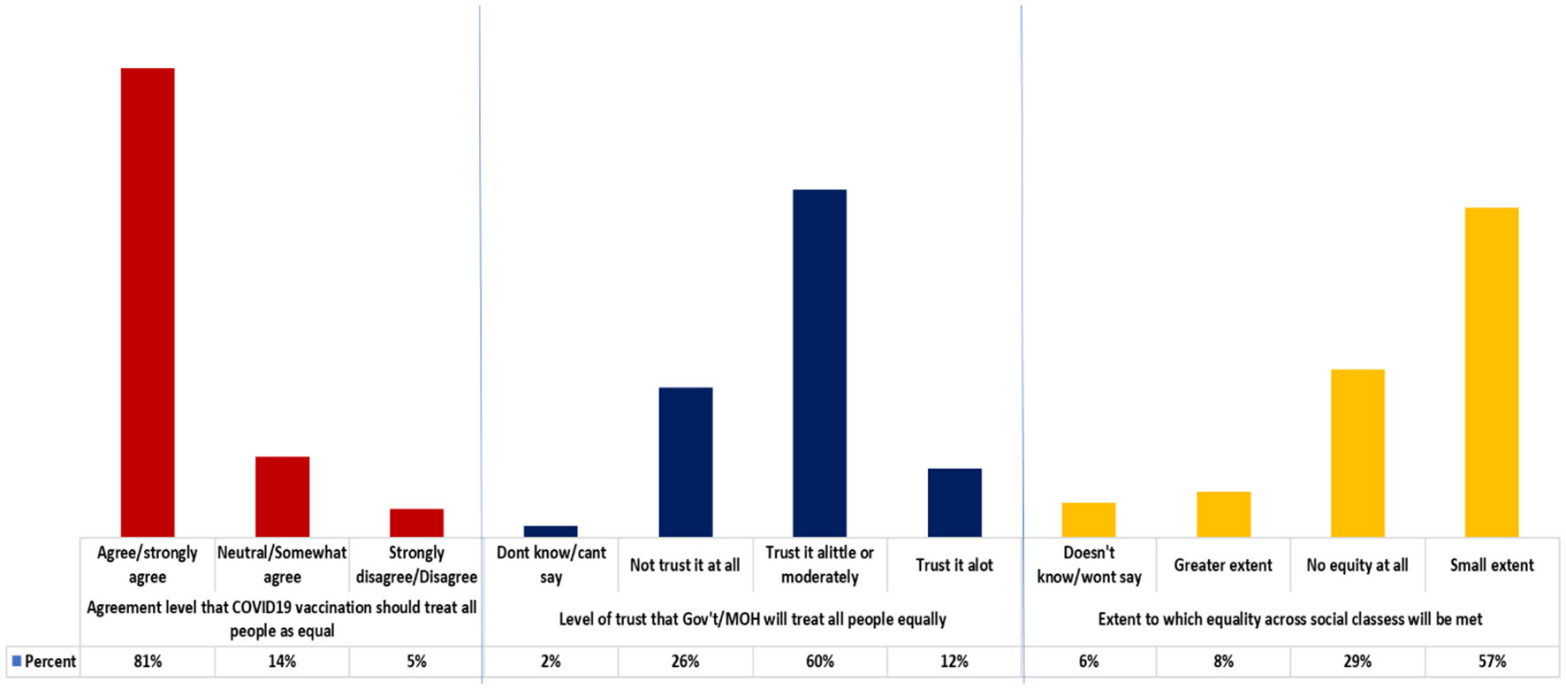
Community feelings, level of trust and perceptions around the equality principle.

The majority of the participants 81.0% agreed/strongly agreed that vaccination should treat all people equally. Most of the participants 60.0% moderately trusted that the government/MOH would ensure that all people are treated equally. More than half of the participants 57.0% agreed to a small extent that equality across social classes will be met (Figure 4). A further analysis shows no variation by gender, participant category, age group and level of education, in the perception that; vaccination should treat all people as equal (mean 4.59, *p* > 0.05), government will treat all people equally (mean 2.29, *p* > 0.05), extent of equality in vaccination across social classes (mean 1.77, *p* > 0.05).

### Qualitative findings

The themes generated included gender and power, economic status, social norms influence, herbal alternatives, and comparative supremacy in tribal identity.

#### Gender and power

Women and children may access vaccines later than men. Whereas, men were against the vaccine, women may be prohibited, especially if their husbands were against it. Respondents reported that men had the upper hand over women. Hence, the vaccination distribution program must consider culture, gender dynamics and power if the equality principle is to be achieved as envisaged.

“*……In our communities, men are considered more superior to women even the decisions made, you have to ask a man or clan members who are men to make the final decision as it is considered culturally appropriate…” (Male local influencer, Eastern region)*.

#### Economic status

Society is constructed along the lines of poor-rich. Thus, the poor think they can only access the vaccine after the rich receive their shots. This attitude may affect the pace of distribution, with the thinking that the poor who are the majority come last.

“*……. everything is about the rich and the poor, the wealthy get everything first, including vaccines, while the poor, who are most of us, are left waiting at the end of the line.” (Female community influencer, Northern region)*

#### Social norms influence

Negative social norms are greatly responsible for hesitancy about immunization programs, and this is no different for the COVID-19 vaccine. Most key informants indicated that social norms around vaccination still prevailed.

“*Patriarchy gives men an upper hand to the detriment of women. Cultural beliefs don't see a man and woman as equal. Therefore, if vaccine distribution in Uganda is to be successful, it is necessary to explore and address particular social norms and their drivers.” (Male community influencer, Western region)*

#### Herbal alternatives

Some participants noted that the community believed that Uganda had not had huge deaths because Ugandans used herbal mixtures that cured COVID-19. Thus, contributing to low uptake of COVID-19 vaccine in rural communities where herbal medicine is more frequently used due to poor access to health services.

“*Rural people in our communities believe in witchcraft and herbal medicine which may hinder progress of vaccination.” (Male special interest participant, Central Uganda)*

### Comparative supremacy driven by corruption and tribal identity

Some participants suggested that Ugandan society is rapidly becoming divided along tribal lines. A large section of community members now believes that they are either superior or inferior to other tribes and thus are either entitled or less/not entitled to services in this case, the COVID-19 vaccine. Deliberate efforts must be undertaken to deconstruct these beliefs.

“*……. if vaccination is to succeed, government should be working for all people no matter the tribe so that they don't feel left out in Uganda.” (Make School Influencer, western region)*

#### Global equity

**Figure 5:**
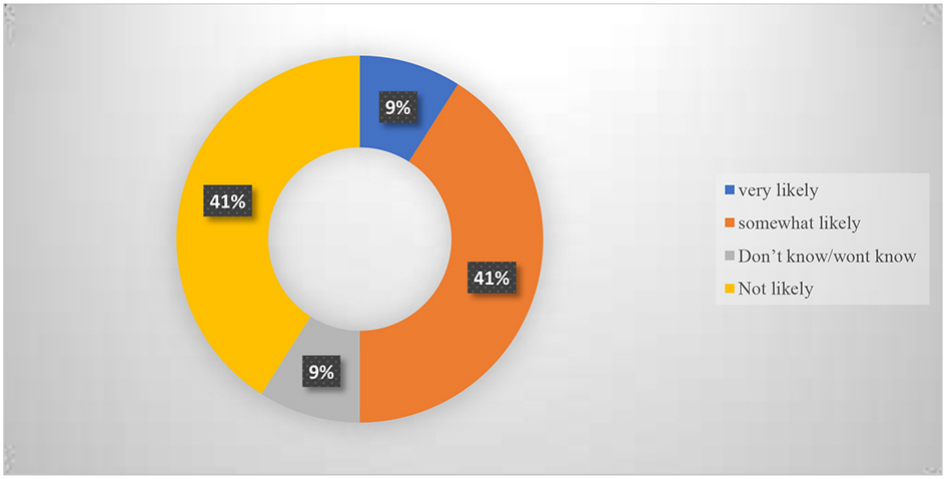
Likelihood that vaccine distribution will ensure equity for LMICs.

Most of the participants 41.0% reported that the COVID-19 vaccine distribution is somewhat likely to ensure equity for low- and middle- income countries (LMICs; Figure 5). Further analysis shows no variation by gender, participant category, age group and level of education on the likelihood that vaccine distribution will ensure equity for LMIC's (mean 1.64, *p* > 0.05).

**Figure 6:**
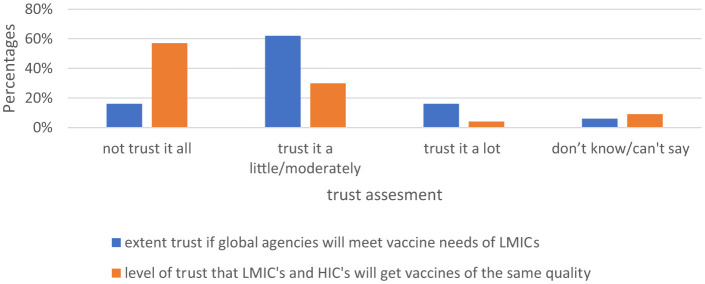
Trust that global agencies including WHO would meet vaccine needs and quality for LMICs.

More than half of the participants 57.0% did not trust at all that LMICs and high-income countries (HICs) would get COVID-19 vaccines of the same quality. Results from further analysis show no variation in the level of trust by gender, participant category, level of education and by age group (mean 1.57, *p* > 0.05). The majority of the participants 62.0% moderately trusted if global agencies would meet vaccine needs of LMICs (Figure 6).

In the spirit of “potential co-production,” the study sought to hear from the community strategies for ensuring that Uganda gets vaccines of the same quality. Table 4 shows the community suggestions that critical stakeholders could adapt for more effective and community-centered vaccine distribution.

**Table 4:** Strategies to ensure that we get vaccines of the same quality.

1	Get Vaccines from trusted countries
2	Ugandan Researchers test vaccines
3	WHO should ensure the vaccine is from one supplier
4	WHO should guide on the vaccines given out
5	Quality control by WHO before allowing vaccines on the market

#### National equity

The majority of participants 86.0% agreed/strongly agreed that vaccine distribution should strongly consider National Equity as shown in Table 5.

**Table 5:** Shows the vaccine distribution based on the national equity.

**Vaccine distribution should strongly consider national equity**	**Freq #**	**Percent**
Agree	9	9%
Disagree	3	3%
Neutral	4	4%
Somewhat agree	7	7%
Strongly agree	77	77%
Total	100	100%

#### Reciprocity

**Figure 7:**
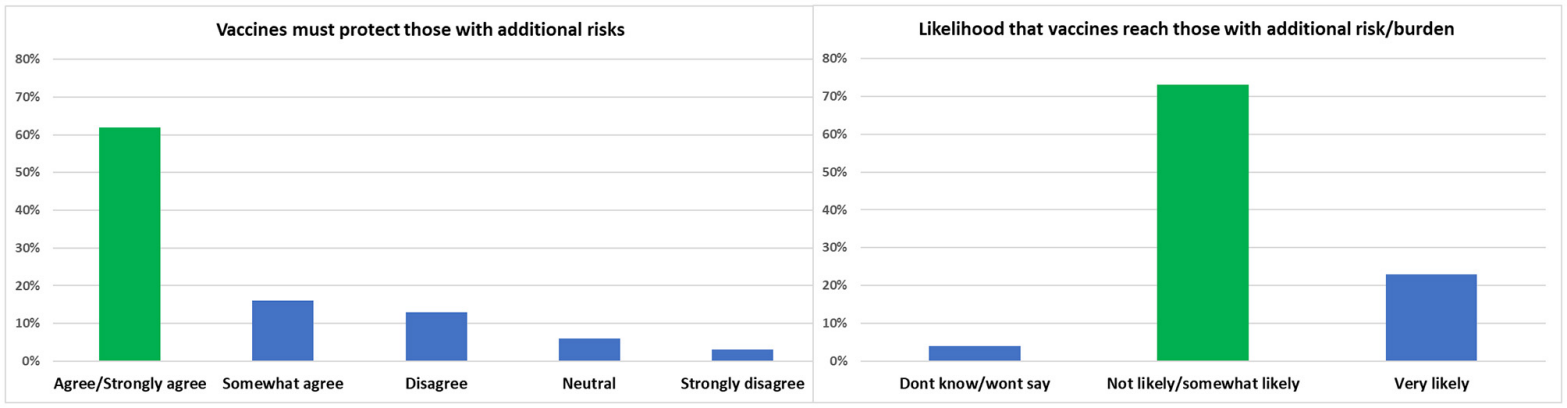
Shows the likelihood that vaccine distribution among those with additional risk.

The majority of participants 62.0% agreed that vaccination should prioritize those with additional risk, and 73.0% did not think that it is very likely that the vaccine distribution would reach those with additional risk faster as illustrated in Figure 7.

#### Legitimacy

**Figure 8:**
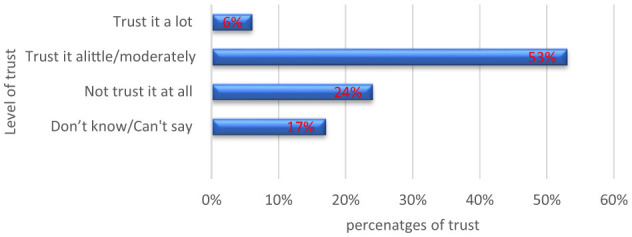
Level of trust that Uganda was engaged in COVID 19 vaccine decisions.

More than half of the participants 53.05% moderately trusted that Uganda was engaged in COVID-19 vaccine decisions (Figure 8).

#### Extent of agreement of fair vaccine distribution

**Figure 9:**
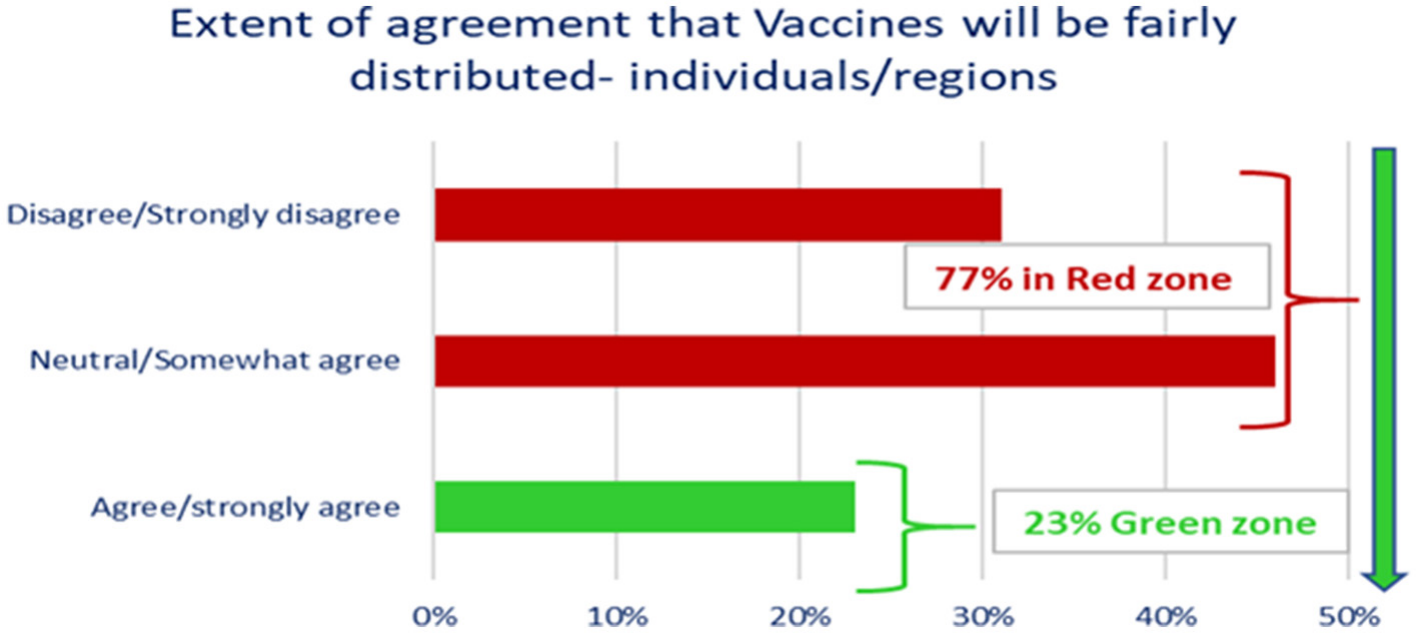
Extent of agreement that vaccines will be fairly distributed to individual/regions.

The majority of participants 77.0% strongly disagreed, or somewhat agreed that vaccines would be fairly distributed among individuals/regions, and there was no variation among participants by gender, level of education, participant category, age group (mean 2.95, *p* > 0.05) as shown in Figure 9.

## Discussion

This mixed-methods cross-sectional descriptive study provides crucial insights into the multifaceted perceptions, knowledge, and attitudes of community influencers in Uganda regarding COVID-19 vaccination. By integrating quantitative and qualitative data, the study uncovers awareness, significant underlying skepticism and mistrust that are vital for informing future public health strategies. This study inquired into community levels of agreement that the COVID-19 vaccine can curb the spread of the pandemic in Uganda. Similarly, several studies indicate that vaccination is vital for protection and can potentially reduce the transmission of infectious diseases (17, 18). The participant characteristics underscore the strategic importance of engaging a diverse array of community influencers. While local community influencers constituted the largest group, the qualitative data highlighted the disproportionate power wielded by political and cultural influencers in shaping behavioral change, despite their smaller representation in the sample. This aligns with diffusion of innovations theory, which posits that opinion leaders, irrespective of their formal roles, are instrumental in the adoption or rejection of new practices within a community (19). Therefore, neglecting the perspectives and influence of these diverse groups can significantly impede public health efforts.

Less than half of the participants knew Uganda/MOH guidelines only. This fragmented awareness underscores the urgent need for a unified and harmonized communication strategy, both globally and nationally, to ensure consistent, clear, and trustworthy information dissemination (20). Inconsistent messaging can breed confusion and erode public trust, as evidenced by the varying levels of guideline awareness observed. A central and concerning finding of this study is the widespread skepticism regarding the COVID-19 vaccine's efficacy belief and trust in curbing the pandemic. A few of the respondents expressed neutrality or some doubt that COVID-19 vaccination could effectively curb the spread of disease. This significant level of skepticism presents a major barrier to vaccine uptake. Additionally, more than half of the participants believed they were likely to acquire the virus even after vaccination, and 27% were unsure. This suggests a critical gap in understanding the primary goals of vaccination, which include preventing severe disease, hospitalization, and death, rather than absolute immunity from infection (21). The analysis indicates no statistically significant differences in beliefs about vaccine effectiveness across participant categories. This uniformity implies that influencers from cultural, health, local community, political, religious, school, and special interest groups hold comparable views, which could be leveraged to promote consistent advocacy for vaccination. This alignment may stem from widespread exposure to national health campaigns or shared media narratives during the pandemic, fostering a baseline acceptance of vaccine utility. Communication strategies must be meticulously crafted to clarify the vaccine's protective mechanisms and its role in mitigating disease severity, directly addressing these prevalent misconceptions to build realistic expectations and foster trust (22).

The community's perspectives on ethical principles for vaccine distribution revealed profound discrepancies between ideal principles and perceived realities with no significant mean differences across categories. There was a slight variation in cultural influencers vs. political influencers and these were not statistically significant. While most participants agreed with the principle of human wellbeing, the pervasive belief that vaccination would not guarantee protection highlights a misalignment between public understanding and scientific evidence, necessitating refined communication about vaccine benefits. This broad agreement underscores a perceived gap in current policy frameworks and supports calls for formalized ethical guidelines to enhance transparency and public confidence. The identified factors undermining equality, poor accessibility, age discrimination, religious biases, social class, corruption, tribalism, and politicization point to deeply entrenched systemic challenges (23). The qualitative themes further elaborated on these concerns, revealing how gender and power dynamics, economic status, negative social norms, reliance on herbal alternatives, and comparative tribal identity significantly influence vaccine access and acceptance. These socio-cultural constructs demand explicit consideration in the design and implementation of equitable vaccine distribution programs. These findings align with global literature emphasizing the role of ethical frameworks in building vaccine trust, particularly in low-resource settings where historical inequities may fuel hesitancy (5).

The principle of global equity also exposed significant distrust. Only few of the participants believed that vaccine distribution would ensure equity for LMICs. Trust in equivalent vaccine quality between LMICs and high-income countries (HICs) was notably low, while moderate trust existed in global agencies. The reasons cited, such as the high cost of vaccines, poor distribution systems, the perceived lack of choice for LMICs, the availability of different vaccine types, corruption, and the preferential access of richer countries, reflect a deep-seated suspicion of global health governance and the fairness of international vaccine allocation (24). The overwhelming distrust regarding the quality of vaccines supplied to LMICs further underscores historical power imbalances and perceptions of exploitation. The community's practical suggestions sourcing from trusted countries, local vaccine testing, and WHO-led quality control demonstrate a strong desire for transparency, accountability, and agency in ensuring vaccine quality. These findings echo critiques of global vaccine nationalism during the pandemic, where LMICs faced delays and inferior allocations, and suggest Uganda could advocate for stronger international mechanisms to ensure parity.

Regarding reciprocity, while more than half agreed that those at additional risk should be prioritized, a majority doubted that this prioritization would translate into practice. This skepticism points to perceived systemic inefficiencies, corruption, and discrimination within Uganda's immunization capacity, mirroring earlier concerns about trust in government and equitable distribution. Effective implementation of reciprocity requires robust, transparent, and equitable targeting mechanisms (25). In addition, the study highlighted a severe crisis of legitimacy. Most of the participants expressed low trust that Uganda was adequately engaged in COVID-19 vaccine decision-making processes. This perceived exclusion significantly erodes confidence in the program's legitimacy, affecting trust in vaccine quality and fairness. The broad disagreement or neutrality regarding fair distribution to individuals and regions further reinforces this lack of trust in governmental processes. Such feelings of disenfranchisement can severely derail vaccination campaigns if not addressed through inclusive governance models and transparent communication about decision-making at both global and national levels (26).

A critical finding from the qualitative component was the identification of key community opinion leaders including religious and cultural leaders, traditional herbalists or healers, youths, celebrities, and local councils who felt excluded from the COVID-19 vaccination campaign and process. These are precisely the informal influencers whose engagement is paramount for overcoming perception, norm, and belief-based barriers to vaccination (27). Their inclusion is essential for designing effective and community-centered strategies. The widespread concern that demand would outstrip supply reflected anxieties about resource scarcity, its potential impact on public health, and the accountability of the system. Indeed, the overwhelming belief in the importance of accountability, juxtaposed with profound distrust in Uganda's accountability due to corruption, political influence, and weak systems, representing a formidable challenge that demands intentional, and systemic reforms.

### Implications, strengths, and limitations

These results have significant implications for public health policy in Uganda and similar contexts. The uniformity in perceptions across categories suggests influencers could be mobilized as allies in promoting ethical vaccine distribution, potentially accelerating uptake through tailored campaigns addressing qualitative barriers like gender dynamics and herbal preferences. Policymakers should integrate community strategies (e.g., WHO quality controls) and explore interventions to dismantle social norms, such as awareness programs on equity and tribal inclusivity. This could enhance vaccine acceptance, particularly in rural areas where herbal alternatives prevail. Strengths of the study include its mixed-methods approach, combining quantitative surveys (*n* = 100) with qualitative insights from diverse influencers, providing a nuanced view of perceptions. The inclusion of multiple categories ensures representation of key societal voices. Limitations include potential selection bias toward influencers, which may not reflect general population views, and small subgroup sizes (e.g., political influencers *n* = 4), limiting statistical power. The cross-sectional design captures perceptions at one point, potentially missing temporal changes post-2021 pandemic phases. Future research could longitudinally assess intervention impacts or expand to non-influencer populations.

## Conclusion

In conclusion, this study paints a complex picture of COVID-19 vaccine perceptions in Uganda. While awareness of guidelines is relatively high, it is overshadowed by pervasive skepticism regarding vaccine efficacy, profound distrust in the fairness and integrity of distribution processes, and a feeling of exclusion from decision-making at global and national levels. Addressing these challenges requires a multi-pronged approach that goes beyond simply disseminating information. It demands transparent, and evidence-based communication tailored to address specific misconceptions about vaccine efficacy, robust accountability mechanisms to combat corruption ensure equitable access, genuine, and inclusive engagement with diverse community influencers. By building trust and fostering a sense of ownership, public health initiatives can navigate these complex social landscapes more effectively and ensure successful outcomes for future public health emergencies. There is strong support for ethical principles in COVID-19 vaccination, addressing equity gaps through culturally attuned strategies is essential for equitable distribution and sustained public trust in Uganda.

## Data Availability

The original contributions presented in the study are included in the article/supplementary material, further inquiries can be directed to the corresponding author.
